# Supplementary material for: Transcription-replication interactions reveal principles of bacterial genome regulation
Source: Res Sq. 2023 Mar 31:rs.3.rs-2724389. Preprint. [Version 1] doi: 10.21203/rs.3.rs-2724389/v1 (PMC10081379; doi:10.21203/rs.3.rs-2724389/v1)
Supplement: 1 [file NIHPPRS2724389V1-supplement-1.pdf]

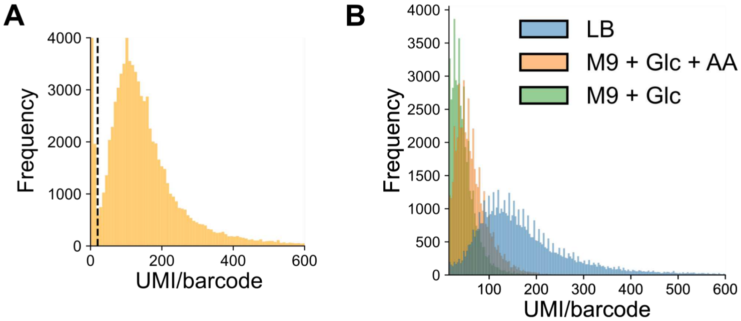

Figure S1: mRNAs captured per cell by PETRI-seq. mRNA captured is quantified as unique molecular identifiers (UMI) per unique cell barcode combination. **A)** *S. aureus* in TSB from Dataset D3. **B)** *E. coli* in different media from Dataset D1.

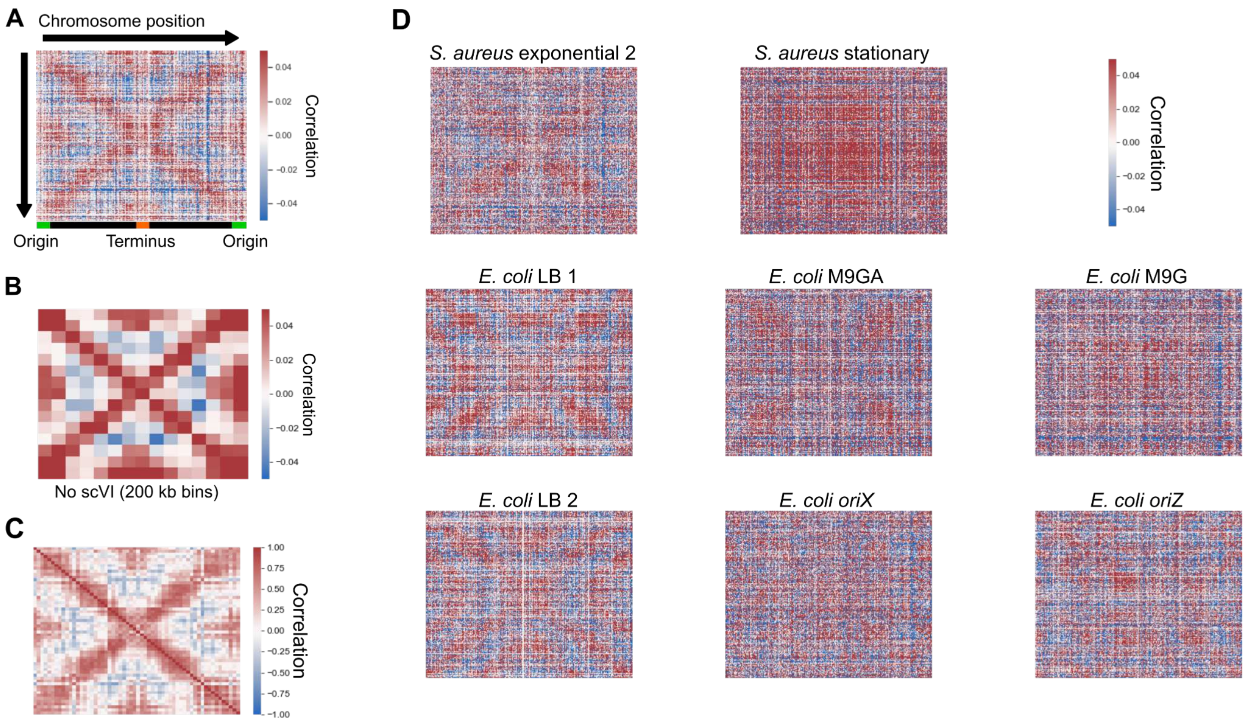

Figure S2: Chromosome-wide gene-gene correlation patterns. A) Spearman correlations from Fig. 1C without binning by chromosome position. **B)** Correlations from Fig. 1C without the use of scVI, binning in 200 kb bins by chromosome position. **C)** Spearman correlations in exponential *S. aureus* data from Dataset D4, averaged in 50 kb bins, as for Dataset D3 in Fig. 1C. **D)** Initial correlations from unbinned, scVI-predicted gene expression data. Sample "*S. aureus* exponential 2" is from Dataset D4, whereas *E. coli* LB replicates 1 and 2 are from Dataset D1 and Dataset D2, respectively.

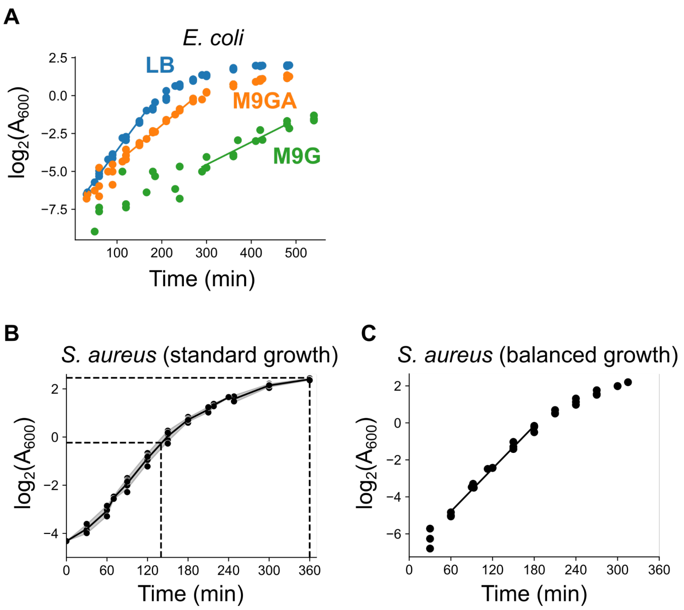

Figure S3: Growth curves of bacterial strains. A) Growth of *E. coli* in three conditions. Doubling times were calculated based on the linear portions of growth (marked as fitted lines). Data are from four (LB and M9GA) or three (M9G) biological replicates. **B)** Growth of *S. aureus* under standard growth conditions. The time and $\log_2(A_{600})$ values when exponential and stationary phase samples were taken are marked with dotted lines. The line is fitted to the mean at each time point, with the gray area representing standard deviation. Data are from five biological replicates. Doubling times for exponentially growing cells are estimated for the linear portion of the curve (~60-150 min). **C)** Growth of *S. aureus* under balanced growth conditions (see Materials & Methods). The black line indicates the linear portion from which doubling time was estimated. Data are from three biological replicates.

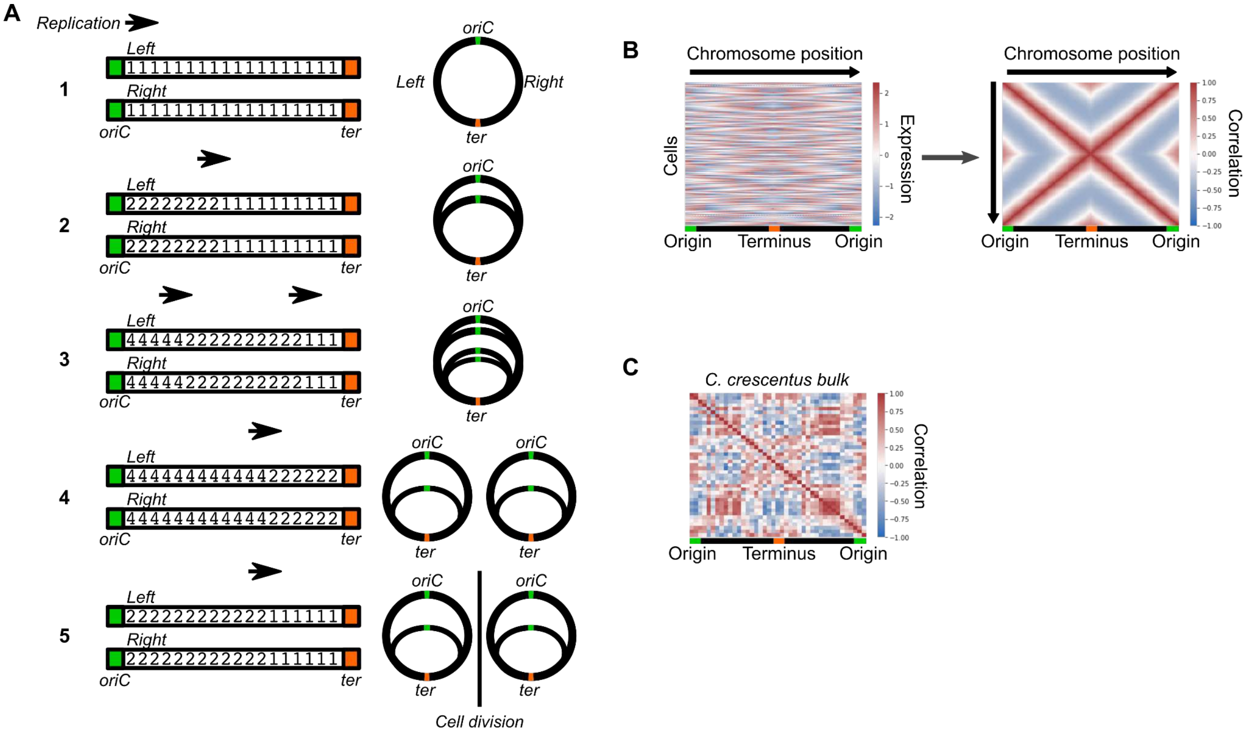

Figure S4: Simulation of replication-dependent gene-gene correlation patterns. A) Schematic figure of the simulation. Each “arm” of the circular chromosome is represented as an array of integers (initially ones), representing each gene. Replication proceeds stepwise from origin to terminus, doubling copy number as it does (steps 1 to 2). At high replication rates, a second round of replication will initiate before the first has finished (step 3). When one round of replication reaches the terminus, that round finishes and after a given time interval copy numbers are globally halved, reflecting cell division (steps 4 to 5). Figures on the right indicate the represented states on the circular chromosome. See Materials & Methods for details. **B)** Simulation of DNA copy number effects predicts the global gene covariance pattern. For 1,000 simulated, unsynchronized cells where the doubling time t_d is equal to the C-period, the normalized, scaled gene expression matrix (*left*) is used to calculate gene-gene correlations (*right*). **C)** Gene expression correlations in synchronized *C. crescentus* bulk RNA-seq from [15]. Scaled gene expression is averaged into 100 kb bins.

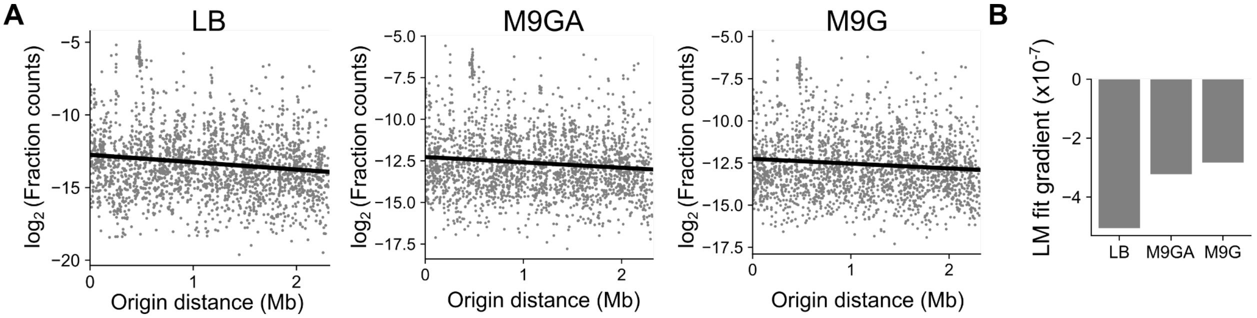

Figure S5: The relationship between origin distance and expression levels. A) For each *E. coli* growth condition, the average fraction of total mRNA UMI from each gene was calculated and $\log_2$-transformed. A linear regression model (black line) was fitted between log-fraction counts and origin distance. **B)** The gradient of the linear model fits in **(A)**. Note that in each case, there is a negative relationship, with a steeper gradient for faster growth rates. This is expected given that at fast growth rates, genes near the origin may attain higher copy number states (>2) than at slow growth rates. Spearman correlations are -0.13 (LB, $P = 3.8 \times 10^{-10}$), -0.09 (M9GA, $P = 2.2 \times 10^{-5}$), and -0.07 (M9G, $P = 6.0 \times 10^{-4}$).

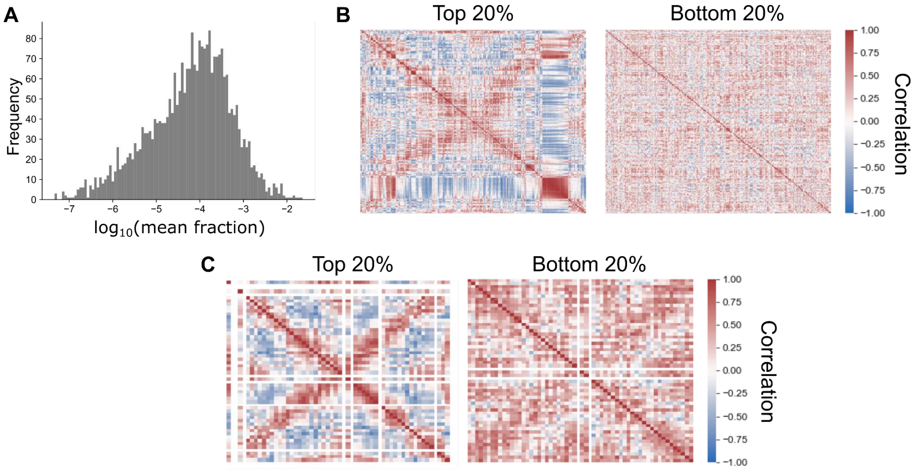

Figure S6: Evidence indicating that the global gene covariance pattern results directly from gene expression. A) Histogram showing that length-adjusted average gene expression varies over several orders of magnitude. This is a broad distribution that would not be expected from genomic DNA. Raw expression counts were normalized by library size (to sum to 1 per barcode) and the average expression was calculated. Length correction was performed as expression divided by gene length then multiplied by median gene length. **B)** Spearman correlations between genes in the top and bottom 20% of genes. Genes are arranged by chromosome order. **C)** Spearman correlations between top and bottom 20% of genes after averaging expression in 50 kb bins as in Fig. 1C. For **(C & D)**, if the pattern was driven by low-level contaminating genomic DNA, it would be expected to be more evident in low-expressed genes (since a higher proportion of reads from these genes should come from genomic DNA) than in high-expressed genes. The opposite is true, with a much stronger pattern in high-expressed genes (presumably due to less noise in these measurements). Taken together, these observations strongly support that the pattern is driven by variation in the transcriptome rather than contaminating genomic DNA.

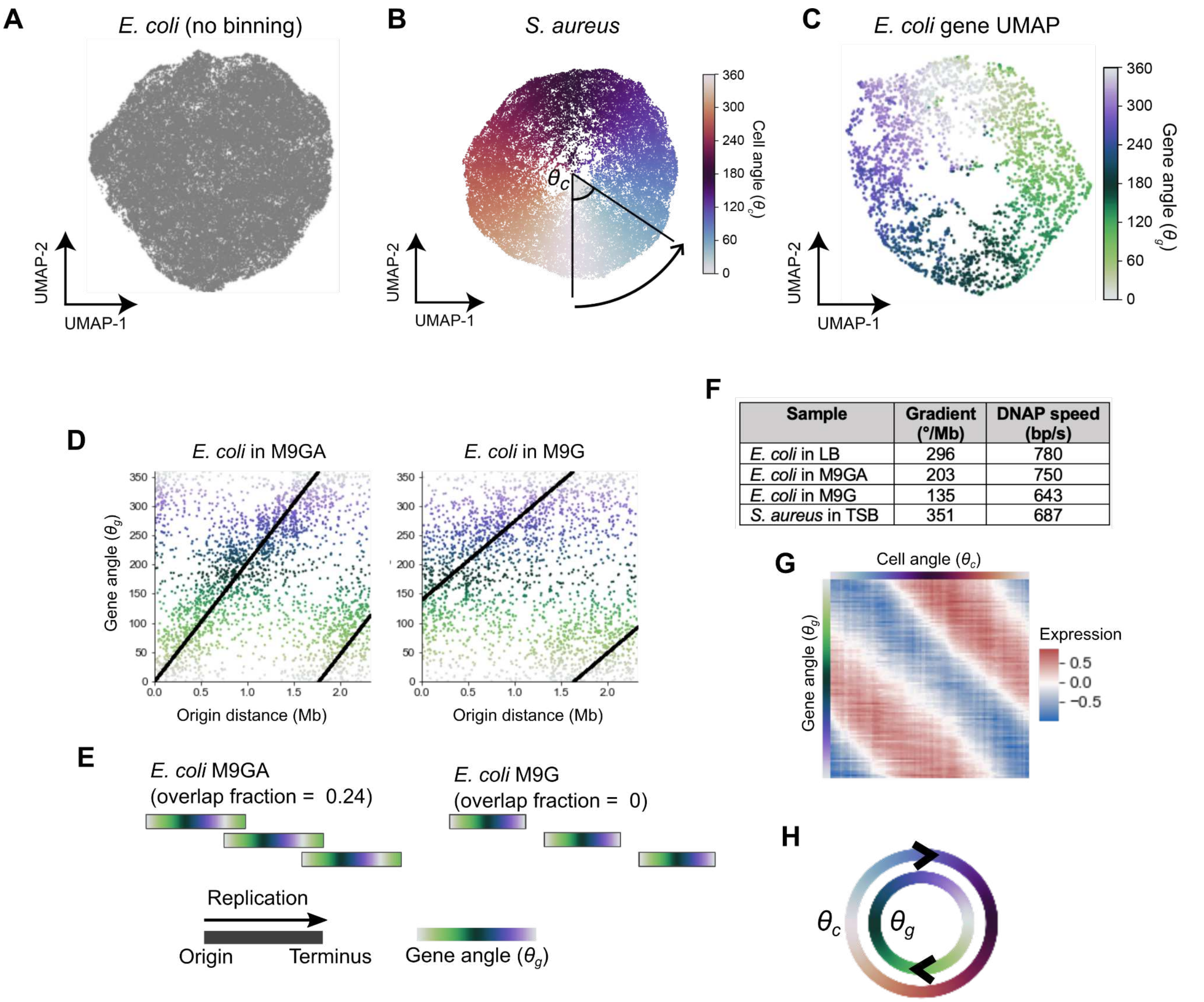

Sample	Gradient (°/Mb)	DNAP speed (bp/s)
E. coli in LB	296	780
E. coli in M9GA	203	750
E. coli in M9G	135	643
S. aureus in TSB	351	687

Figure S7: Cell and gene angle analysis to model replication-dependent gene expression. **A)** UMAP analysis of LB-grown *E. coli* based on scVI-predicted expression. **B)** UMAP of *S. aureus* with gene expression averaged in 50 kb bins by chromosome position. Cells are colored by the cell angle θ_c between UMAP dimensions relative to the center of the projection. **C)** UMAP of *E. coli* genes, performed on the same data as the PCA in Fig. 2D. Gene angles shown are those derived from PCA. **D)** The relationship between θ_g and origin distance for *E. coli* grown in M9 + glucose + amino acids (M9GA) or M9 + glucose (M9G). The black line indicates the model fit as described in Materials & Methods Section "Modeling the gene angle-origin distance relationship". **E)** Predicted replication patterns as for Fig. 2G but for *E. coli* under slower growth conditions. **F)** Gradients of the gene angle-origin distance relationship and estimates of DNA polymerase speed from these gradients. See Materials & Methods for details. **G)** Expression in LB-grown *E. coli* is first averaged in 100 bins by θ_c then averaged in 100 bins by θ_g to yield the 100 x 100 matrix represented here as a heatmap. This is used to train the model to predict gene expression at a given point in the cell cycle (θ_c) for a given gene (θ_g). **H)** Conceptual representation of the cell cycle expression parameterization. Cells are ordered in their cell cycle state by θ_c, whereas genes are ordered by their cell cycle expression by θ_g. Cell cycle expression can be described as the concurrent cycling of cells and genes ordered by these metrics.

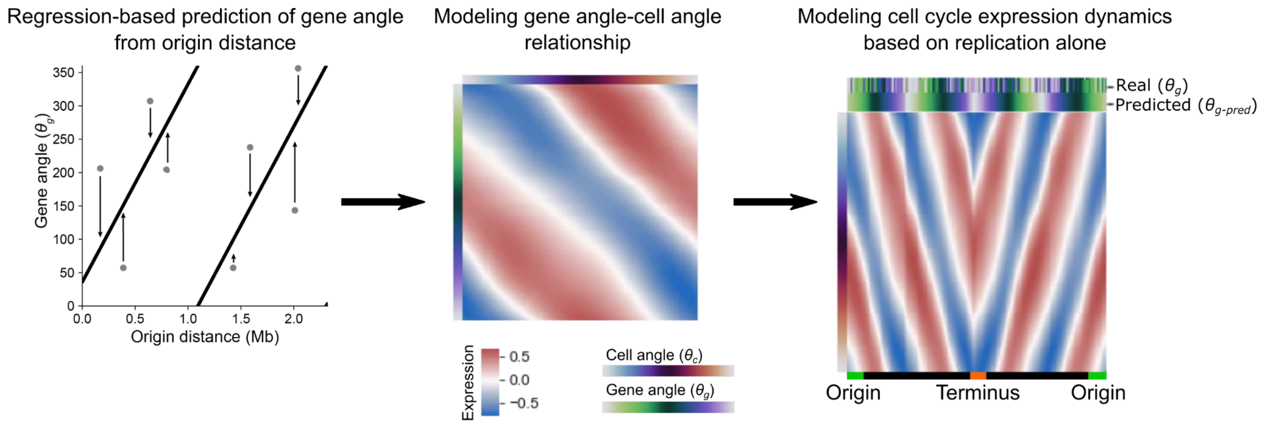

Figure S8: Predicting gene expression dynamics based on distance from the origin. The following pipeline predicts cell cycle expression for a given gene based only on its distance from the origin of replication. A regression model predicts gene angle $\theta_{g\text{-}pred}$ based on origin distance alone (*left*) and this is converted into a prediction of expression by cell angle θ_c using a second regression model (*middle*). Ordering genes by chromosome position (*right*) shows a smoothed version of the expression pattern in Fig. 2B. The bar at the top of this figure shows the real and predicted gene angles. Data are from *E. coli* grown in LB. See Materials & Methods for full details.

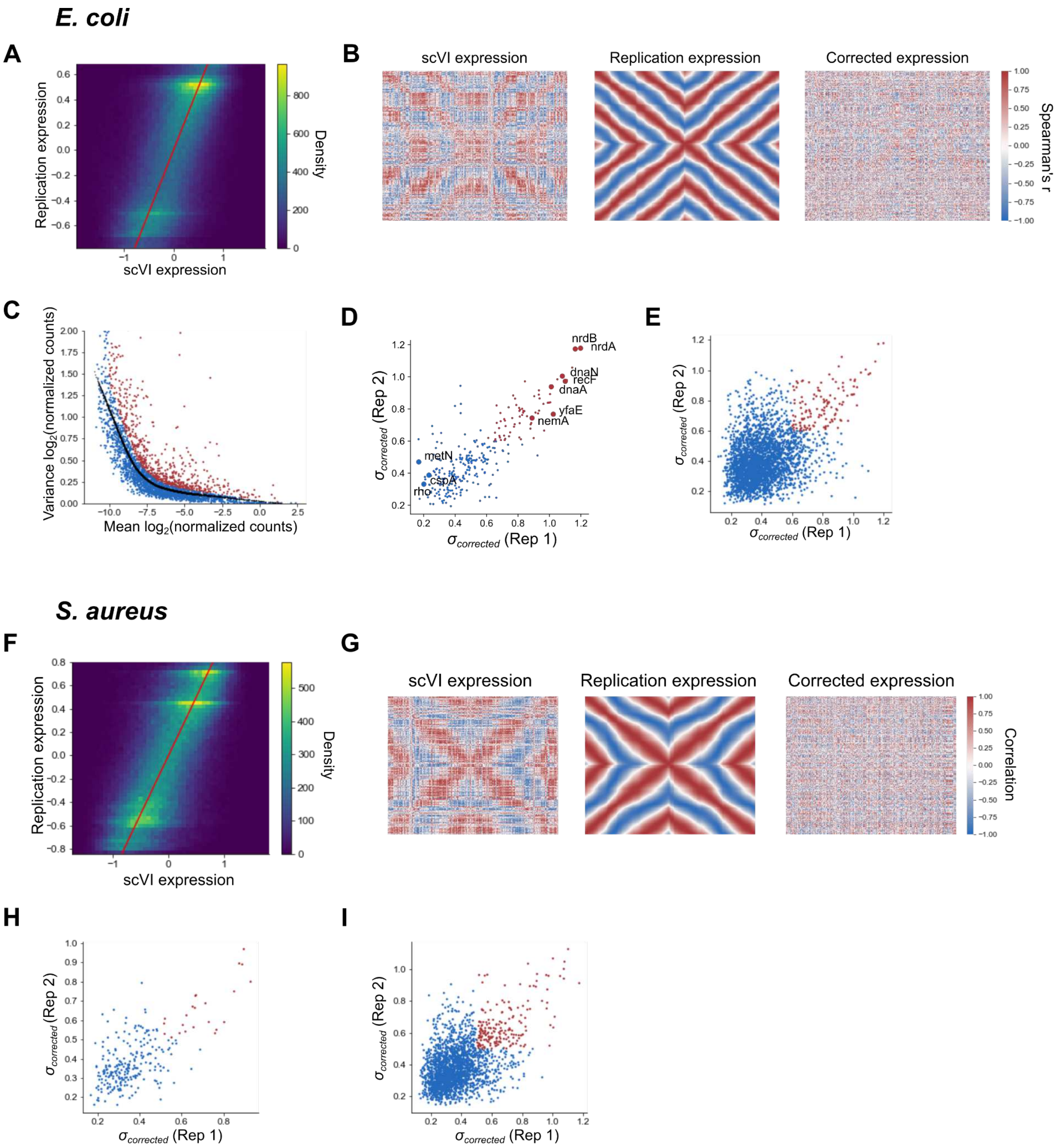

Figure S9: Correcting for and measuring divergence from predicted replication-associated patterns. A) Two-dimensional histogram for *E. coli* showing the relationship between observed expression from scVI and replication-predicted expression. Expression is averaged in 100 bins by cell angle θ_c. The red line indicates $x = y$ i.e. the case where expression in both matrices is identical. Overall, there is a rough 1:1 correspondence between observed and predicted expression, indicating a good model fit. **B)** Gene-gene correlations in LB-grown *E. coli* across θ_c-binned expression data (100 bins) for the full scVI observed model (*left*), the replication-only model (*middle*), and the corrected model that is the difference of the two expression matrices (*right*). **C)** The mean-variance relationship in *E. coli* of log-transformed normalized counts. The black line indicates the locally weighted scatterplot smoothing (LOWESS)-fitted values and red points are genes classed as highly variable. See Materials & Methods for further details. **D)** Comparison of the divergence score $\sigma_{corrected}$ between LB-grown *E. coli* in Datasets D1 & D2 of genes classed as highly variable in both datasets (287 genes). Red indicates replication-divergent genes ($\sigma_{corrected}$ > 0.6). **E)** Comparison of $\sigma_{corrected}$ (standard deviation of divergence from the replication model) between LB-grown *E. coli* in Dataset D1 and

Dataset D2 of all genes present in both datasets. Red indicates $\sigma_{corrected}$ > 0.6 in both datasets, meaning that they are considered replication-divergent. The Pearson correlation between replicates is 0.38. **F)** Two-dimensional histogram as in **(A)** but for *S. aureus*. **G)** Gene-gene correlation plots as for **(B)** but for *S. aureus*. **H & I)** Comparison of $\sigma_{corrected}$ (standard deviation of divergence from the replication model) between *S. aureus* in Dataset D5 and Dataset D6 for highly variable genes in both datasets **(H)** (Pearson's r = 0.66) and all genes **(I)** (Pearson's r = 0.48). Red indicates $\sigma_{corrected}$ > 0.5 in both datasets, meaning that they are considered replication-divergent.

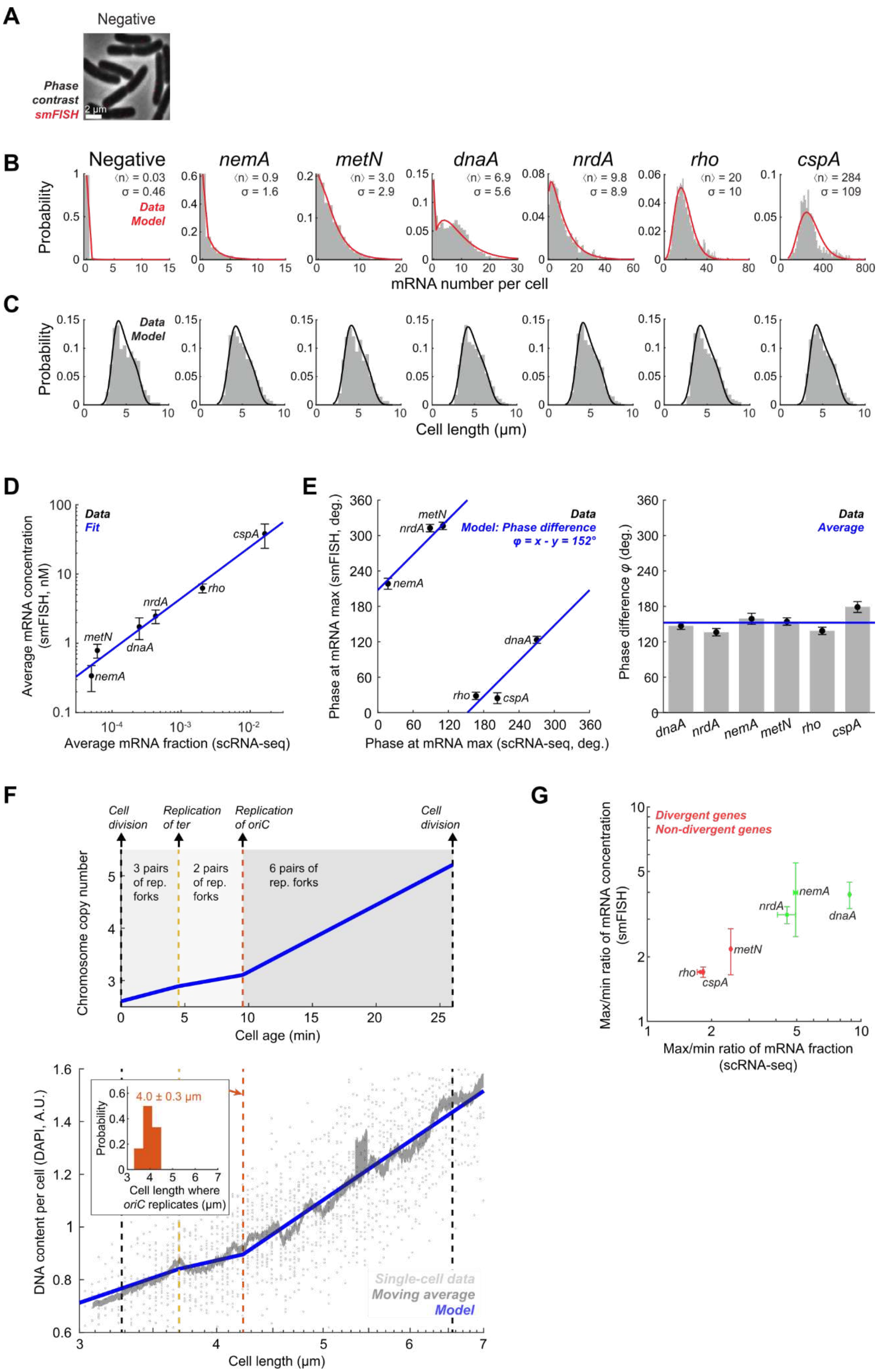

Figure S10: smFISH analysis of cell cycle gene expression correlates with phase-shifted scRNA-seq data. A) Negative control for smFISH labeling. *E. coli* cells labeled against bacteriophage lambda *cI* mRNA. smFISH signal is shown using the same contrast as in Fig. 3,

B & D. See Section “smFISH”. **B)** The distribution of mRNA copy-number per cell for each gene. See Section “mRNA quantification”. Red line, fit to a negative binomial distribution plus a “zero spike”[10]. **C)** The distribution of cell length in each sample. Black line, fit to the theoretical model of [87], see Section “Modeling the distribution of cell length”. **D)** Comparison of the population-averaged mRNA fraction, as measured using scRNA-seq, with mRNA concentration, as measured using smFISH. Markers and error bars indicate mean ± SD from two datasets of each method. Blue line, fit to a function $y = ax^b$. **E)** Estimation of the cell-cycle phase difference between scRNA-seq and smFISH. The phase of each dataset was estimated as described in Section “Cell-cycle analysis of mRNA concentration”. *Left*, markers and error bars indicate mean ± SEM from two datasets of each method. Blue line, fit to a linear function, indicating a constant phase difference φ. *Right*, the estimated phase difference across the six genes examined. **F)** Top, the theoretically predicted cellular DNA contents as a function of cell age, see Section “Inferring cell-cycle phase from the DAPI signal”. Bottom, DAPI-measured DNA content per cell as a function of cell length. Single-cell data was binned based on cell length (moving average ± SEM, 21 cells per bin), Blue line, fit to the theoretical model. Inset, the distribution of the inferred cell length where *oriC* replicates, estimated from all smFISH samples. **G)** Divergent genes exhibit a larger amplitude of cell-cycle fluctuations. The ratio between the maximum and minimum expression level of different genes, as measured using scRNA-seq and smFISH. The mRNA fraction (scRNA-seq) and concentration (smFISH) were obtained as in Fig. 3 B & D, 2nd and 3rd columns. The maximum and minimum levels were determined from the binned data. Markers and error bars indicate mean ± SD from two datasets of each method.

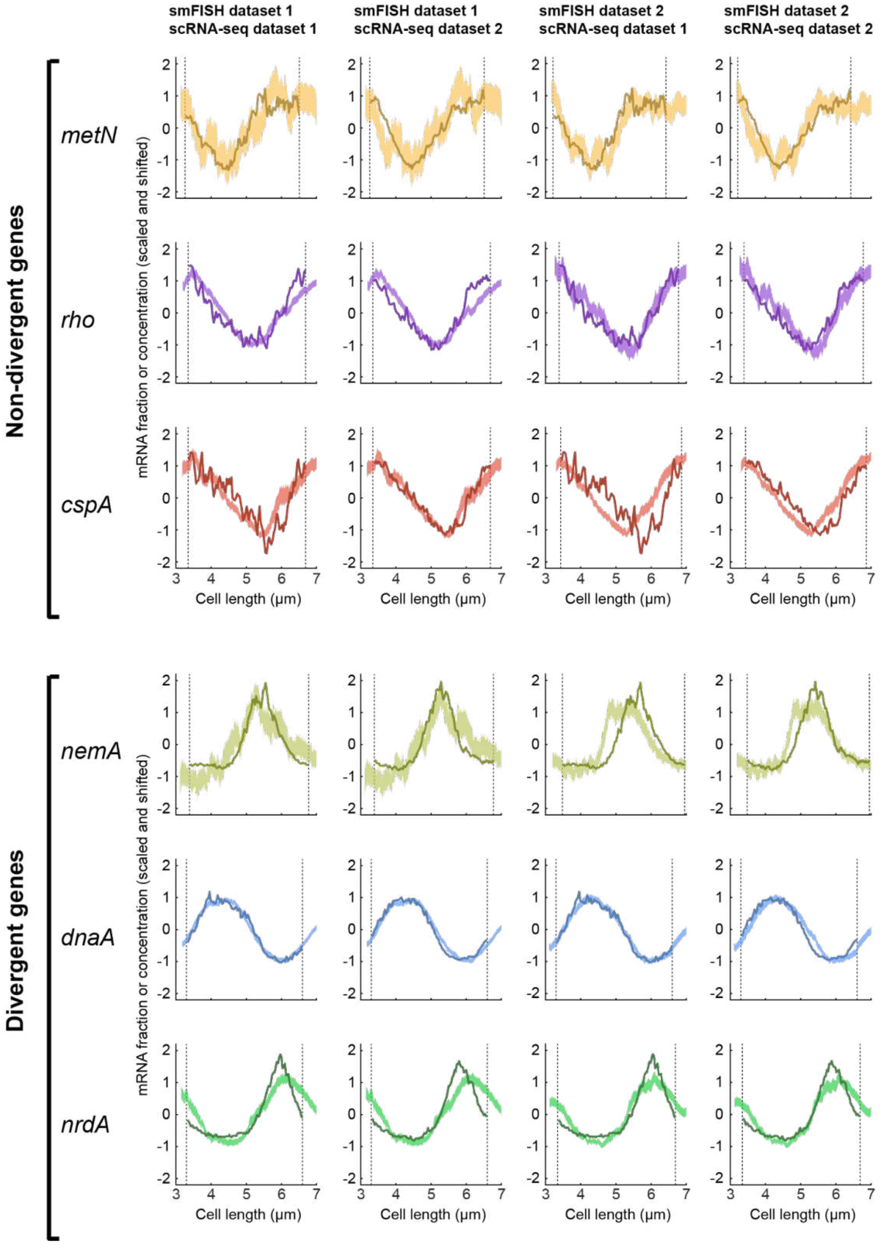

Figure S11: Cell cycle analysis of smFISH and scRNA-seq shows good agreement across biological replicates. Pairwise comparison between two smFISH and two scRNA-seq datasets. Analysis as in Fig. 3B & D, 4th column. See Section “Cell-cycle analysis of mRNA concentration”.

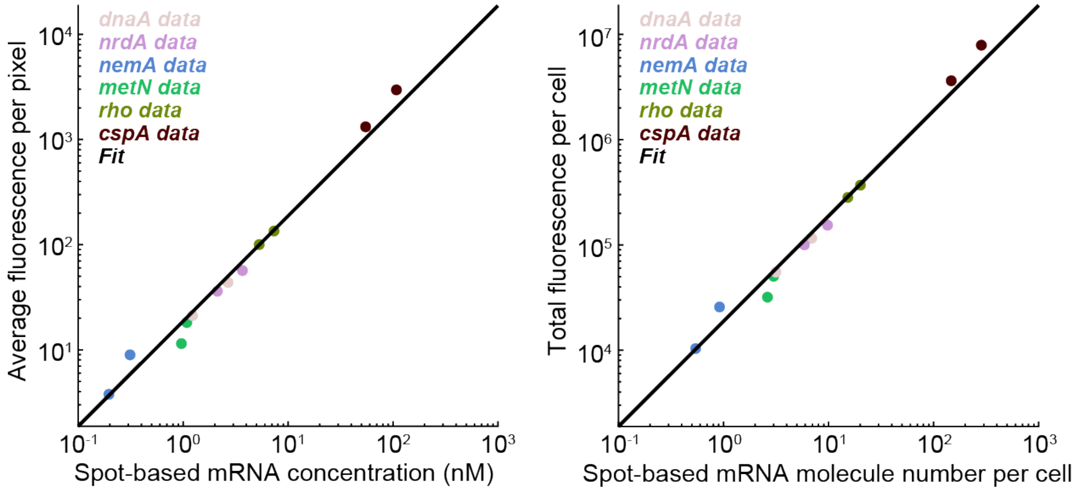

Figure S12: Consistency between spot-based and cell-based smFISH quantification. Comparison of the mRNA levels inferred from smFISH data using spot-based and cell-based mRNA quantification. Both methods are described in Section "mRNA quantification". *Left*, mRNA concentration. *Right*, mRNA copy number per cell. Markers indicate mean values from each smFISH sample (Error bars are smaller than marker size). Black line, fit to a linear function.

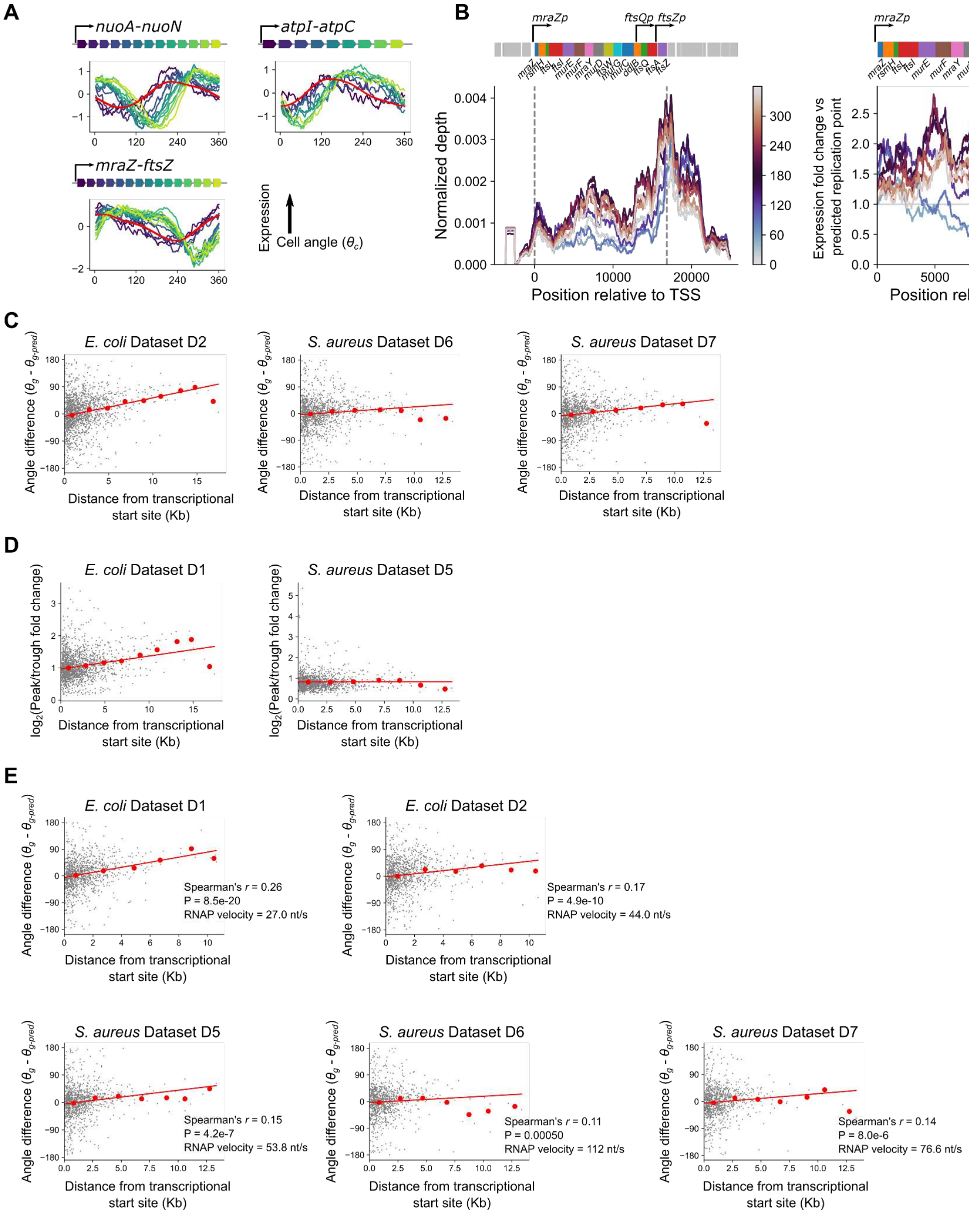

Figure S13: The relationship between distance from the transcriptional start site and gene expression timing and amplitude. A) Cell cycle gene expresion plots for operons showing "delayed" genes as in Fig. 4B but for LB-grown WT *E. coli* from Dataset D2. The red line indicates predicted expression. **B)** Normalized per-base read depth at the *mraZ-ftsZ* locus. *Left*: Normalized expression as in Fig. 4D. *Right*: Fold-change relative to expression at the predicted time of replication, as in Fig. 4E. Schematic figures of the locus depict a simplified version since several internal promoters have been identified. **C)** Plots of maximum distance from a transcriptional start site against difference between predicted and observed angles as in Fig. 4C. Red line indicates the linear model fit and red points indicate averages of 2 kb bins. Data are shown for additional *E. coli* and *S. aureus* replicates. **D)** Plots as in **(C)** but of maximum distance from a transcriptional start site against the $\log_2$-transformed peak/trough ratio in gene expression, calculated as described in Materials & Methods. **E)** Plots as in **(C)** but using manual operon annotation. Here, any tandem, contiguous stretch of genes with an intergenic distance less than 40 bp is considered an operon. Transcriptional start sites are defined as the start position of the first gene in the operon.

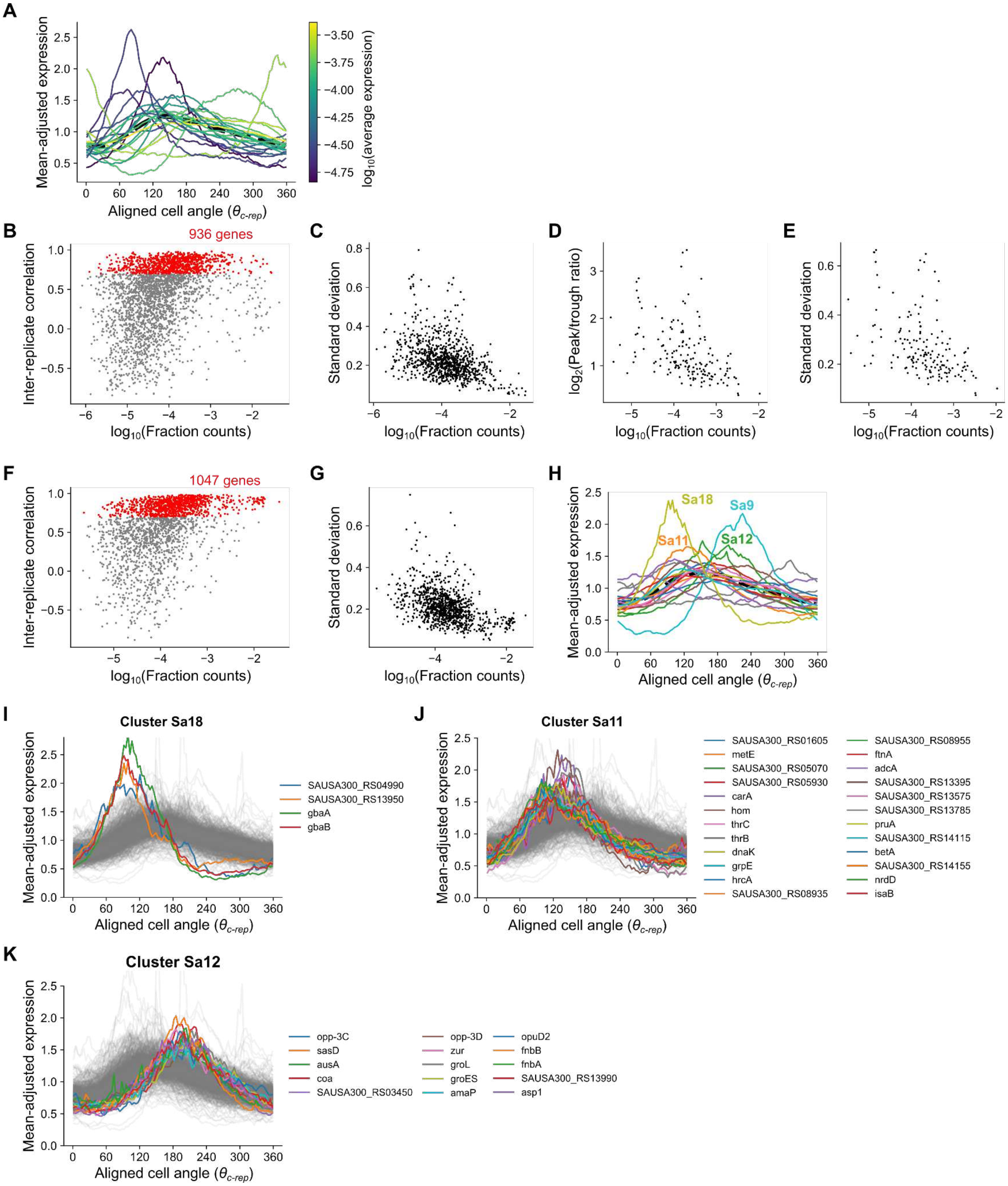

Figure S14: Expression-amplitude relationships and *S. aureus* cluster profiles. A) Clusters as in Fig. 5B but colored according to their average, length-corrected expression. This was determined by a gene's mean fraction of total mRNA that was length-corrected by dividing by its length and multiplying by the median gene length across genes. **B)** Scatter plot of length-corrected mean fraction counts (i.e. fraction of a gene within the whole transcriptome) against Spearman correlation in *E. coli*. Spearman correlations for each gene were calculated as the inter-replicate correlation between cell cycle gene expression measurements averaged in 100

bins by θ_c (replicates from Datasets D1 & D2). Red genes indicate the reproducible genes used in Fig. 5. **C)** Length-corrected mean expression against standard deviation across expression averaged in 100 bins by θ_c. **D & E)** Plots as in Fig. 5E and **(C)** but including only those genes with Spearman R > 0.9 (instead of 0.7). **F)** Plot as in **(B)** but for *S. aureus* (replicates from Datasets D5 & D6). **G)** Plot as in **(C)** but for *S. aureus*. **H)** Plot as in Fig. 5B except for mean expression of *S. aureus* clusters. Genes situated on mobile genetic elements were removed prior to clustering analysis. **I-K)** Plots of individual genes from clusters indicated in **(H)**.

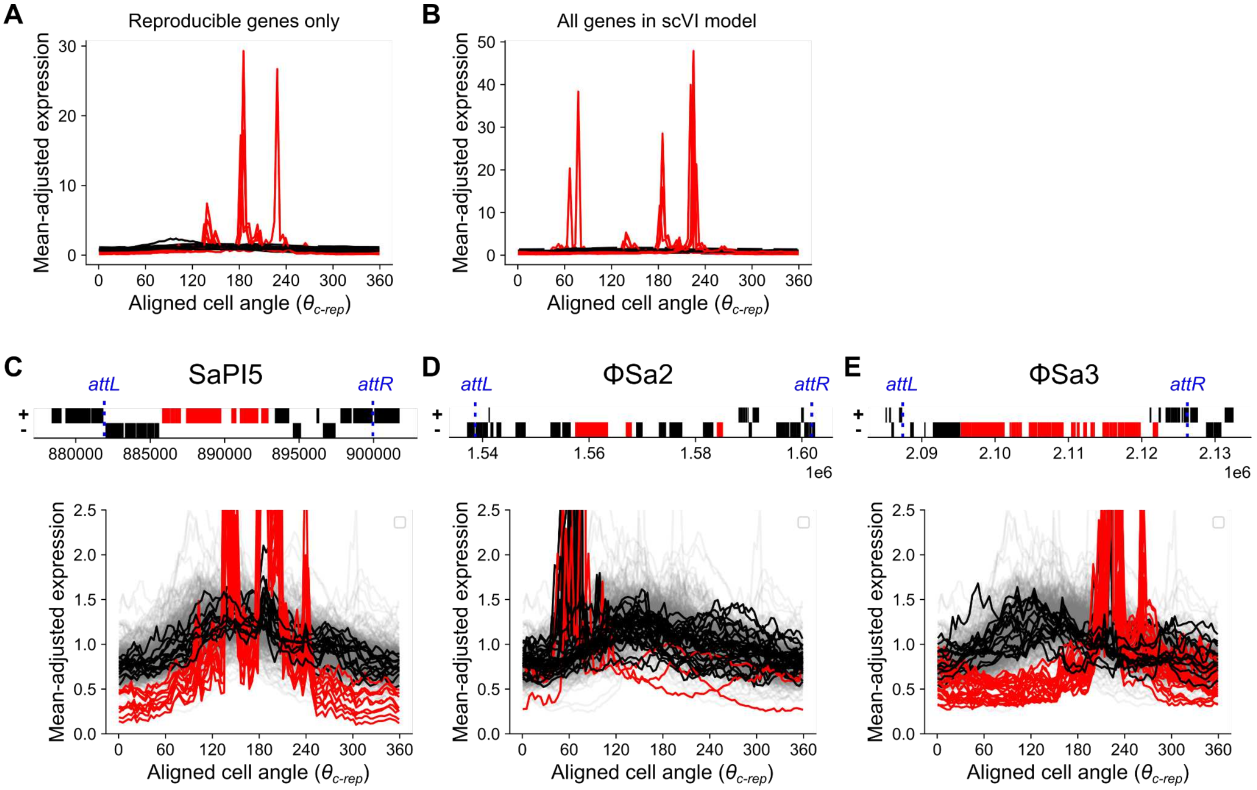

Figure S15: Core genes of mobile genetic elements show highly divergent expression patterns. A) Mean cluster expression of reproducible genes partitioned into 20 clusters by aligned gene expression ($\theta_{c\text{-}rep}$). Clusters in red are those that only contain genes located within MGEs. **B)** Plot as in **(A)** but with clustering performed on all genes included in the scVI model (regardless of reproducibility). These cluster assignments are used for **(C-E)**. **C-E)** Expression of genes within mobile elements. Genes are colored based on whether they are in MGE-exclusive clusters from **(B)** (red) or other clusters (black). *Top*: schematic figure of MGE gene content. The *x*-axis represents chromosomal coordinate and + and - strands are plotted separately by *y*-axis position. Predicted attachment sites *attL* and *attR* denote the predicted boundaries of the MGE and are annotated in blue. Annotation for MGEs was taken from the online tool Phaster[92]. *Bottom*: Plots of MGE genes by aligned gene expression ($\theta_{c\text{-}rep}$) as represented in Fig. 5. Gray genes represent the non-MGE background. Note that phage ΦSa2 is disabled and expression of its MGE-specific ("red") cluster genes is low (0.002% of cells contain at least three transcripts) compared to the staphylococcal pathogenicity island (SaPI) 5 (0.4%) and phage ΦSa3 (0.07%), potentially contributing to the less clear delineation between expression profiles by gene type. MGE-specific expression patterns may arise due to MGE mobilization and these patterns may represent rare events that are not effectively captured by our cell cycle analysis, meaning that the plots here should be interpreted with caution.

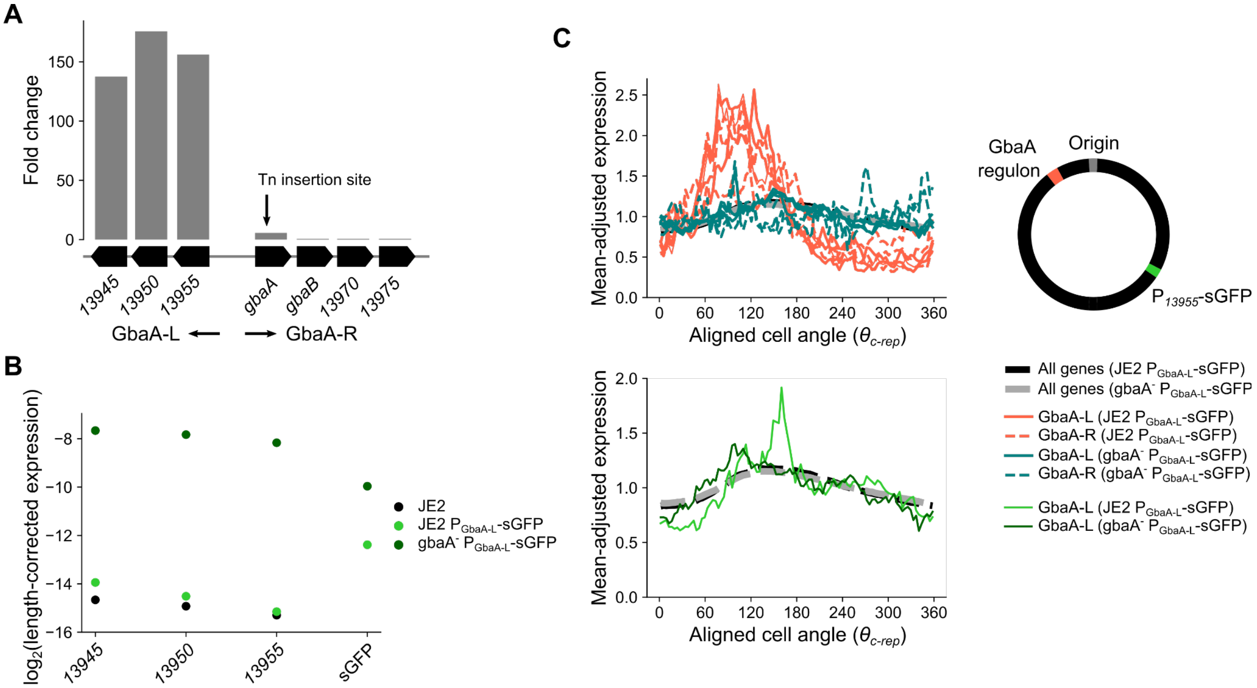

Figure S16: Effects of gbaA disruption on cell cycle gene expression. A) Expression fold change of genes in the GbaA regulon after *gbaA* transposon insertion. Genes of the GbaA-L operon increase in expression >100–fold. However, due to the location of the transposon insertion towards the 5' end of *gbaA*, induction of GbaA-R genes is not observed. Genes with names starting with *SAUSA300_RS* are truncated to give only the unique number. **B)** Average expression of GbaA-L genes and sGFP in reporter strains (compared to JE2 in measurements from the same experiment). Average expression measured as fraction of total mRNA was length-corrected as elsewhere by dividing by the gene length and multiplying by the median gene length across all genes. Note that sGFP expression in JE2 P_{GbaA-L}-sGFP is approximately fourfold higher than that of GbaA-L genes, and the derepressed form in *gbaA*⁻ P_{GbaA-L}-sGFP is also fourfold lower (possibly reflecting lower copy number due to its further distance from the origin). Therefore, while repression of the GbaA-L locus is ~96-fold, repression of sGFP by GbaA is only 5.3-fold. **C)** Comparison of aligned expression (θ_{c-rep}) (as in Fig. 5) for GbaA regulon genes and sGFP in the two reporter constructs. Thick black and gray lines represent average expression across all reproducible genes. The schematic figure represents the relative positions of the GbaA regulon and the P_{GbaA-L}-sGFP integration site.

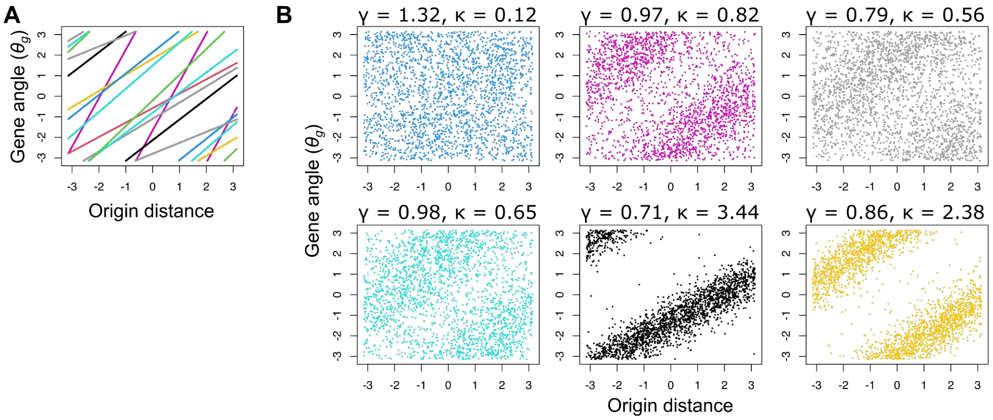

Figure S17: Sampling from the prior of the gene angle-origin distance regression model. Based on the model and priors specified in Materials & Methods, values were randomly sampled from the prior and used to predict either the expected gene angle *A* **(A)** or the predicted value of gene angle θ_g after von Mises sampling **(B)**. For each sampled set of parameters in **(B)** the gradient γ and concentration parameter κ are shown. Both θ_g and origin distance *D* are standardized to the range -π to π as per the model requirements. Overall, the prior assumptions of the model are that there is a positive, linear relationship between θ_g and *D*, but there is considerable flexibility regarding the gradient (and hence degree of wrapping), value of θ_g at D = 0, and noise.

Table S1: Information about datasets and samples used. A_{600} refers to the optical density at the time of harvesting. *Growth *E. coli* MG1655 in LB was measured in a separate series of experiments for each dataset.

Dataset	Sample	Strain	Medium	A_{600}	Doubling time (min)	# cells	Median mRNA UMI/barcode
D1	eco_lb_1	*E. coli* MG1655	LB	0.15	26.0 ± 1.3 (n = 4)*	57,627	152
	eco_mga_1	*E. coli* MG1655	M9GA	0.185	39.4 ± 2.3 (n = 4)	50,920	56
	eco_mg_1	*E. coli* MG1655	M9G	0.062	69.1 ± 9.8 (n = 3)	45,898	40
D2	eco_lb_2	*E. coli* MG1655	LB	0.152	27.0 ± 1.6 (n = 4)*	69,396	93
	eco_orix_1	*E. coli* MG1655 ΔlacIZYA oriX-<>[25,27]	LB	0.127	27.2 ± 2.4 (n = 4)	25,967	97
	eco_oriz_1	*E. coli* MG1655 ΔlacIZYA oriZ-<>[26]	LB	0.14	26.6 ± 2.1 (n = 4)	32,151	100
D3	sau_tsb_1	*S. aureus* USA300 LAC	TSB	0.97	30.1 ± 0.8 (n = 5)	73,053	135
D4	sau_exp_plus	*S. aureus* USA300 LAC	TSB	1.12	30.1 ± 0.8 (n = 5)	13,075	87
	sau_exp_minus	*S. aureus* USA300 LAC	TSB	1.12	30.1 ± 0.8 (n = 5)	8,182	57
	sau_stat_plus	*S. aureus* USA300 LAC	TSB	5.76	NA	40,772	27
	sau_stat_minus	*S. aureus* USA300 LAC	TSB	5.76	NA	15,122	24
D5	sau_wt_1	*S. aureus* USA300 LAC	TSB	0.088	24.9 ± 0.6 (n = 3)	49,307	159
D6	sau_wt_2	*S. aureus* USA300 LAC	TSB	0.112	24.9 ± 0.6 (n = 3)	38,426	136
	sau_je2_1	*S. aureus* JE2	TSB	0.107	NA	46,719	107
	sau_gbaa_1	*S. aureus* JE2 *SAUSA300_2515::bursa* (Nebraska library # NE355)[93,94]	TSB	0.103	NA	37,985	109
D7	sau_wt_3	*S. aureus* USA300 LAC	TSB	NA	24.9 ± 0.6 (n = 3)	31,852	152
D8	sau_je2_2	*S. aureus* JE2	TSB	NA	NA	21,006	210
	sau_je2_pgbaal_1	*S. aureus* JE2 pJC1111-$P_{GbaA\text{-}L}$-*sGFP*	TSB	NA	NA	17,206	250
	sau_gbaa_pgbaal_1	*S. aureus* JE2 *SAUSA300_2515::bursa* pJC1111-$P_{GbaA\text{-}L}$-*sGFP*	TSB	NA	NA	13,420	225

Table S2: DNA oligos used for smFISH

Gene	Number of probes	Probe sequences (5' - 3')		Source
dnaA	24	TGCCAAAGCGAAAGTGACAC<br>AATTCTGTGGCTGGTAACTC<br>CAATGGGCGTATCCACATAC<br>AGCGTGTTATCGCTCAGTTC<br>GCAGAAACTGGTTAGCAGTC<br>CGACTTCAAAACGCAGCTGT<br>AGAACGATAGGTCGGTTCTG<br>ACGTGTGTTTGACGTTTACG<br>CGCCAGTTGGTTAGATTTAC<br>ATGCAGCAGGTGAGTTTTAC<br>TAAACCACTTTGGCATTCGG<br>TTTGCAGGGCTTTAACCATG	ATCTACGGAACGGTAGTAGC<br>GAATATCGTCGATCAGCAGT<br>GGCGTTGAAGGTGTGGAAAA<br>ATAGCGATCCGAGGTGAGAA<br>CAACGCCGTTGATCTCTTTC<br>TTTTTCATCAGGATCGCCAC<br>ACGAATGTCGTTTTCGTCGG<br>GTACGTTAGATCGTAGACGC<br>GGTAAAGTTGGCATTGGCAA<br>CCGTCTTCTGAATATTGTCG<br>CGCGACTTTGATCTTGTAGT<br>TGTGGTTAGTCAGCTCTTTC	This work
nrdA	24	CAATCCAGAACGCGATGGAT<br>AAACTGAATGTGGGAGCGCA<br>ATGTCAGAGGTCTTGATACC<br>CAGCCTTGATAATGGTTTCG<br>CGCGGCGAGATACTGATAAT<br>TTTACGCAGGTGGAAGATCG<br>ATCTCGACCATTTTCACCAC<br>GAACTCTTCTTCCGTGTAGT<br>CGATAAAGGTGTCCATCTGC<br>AAGGTCATATCACGGTCGTG<br>CTGCTTAACGGCAGCATAAG<br>ATATAAAGGAACTGGGCGCT	GGGTAGTTCGAGAACAAGCA<br>ATATTGCAGGCGCGTTTCAC<br>AACCGCGTCGTAAAAACGCT<br>CGTCGGCAGCGAAATTTTAA<br>TTGATGGAATCCAGGCTGTC<br>TTGTAGAACGGAATGCAGCC<br>TTTCACCGCTGTCTGGAAAT<br>CGGTTGTTTTTCAACACCAG<br>CTTTCAGCAGACGGGTATAC<br>GCTGAACAGGGTGATATCTT<br>GACGTTCAAACTCTTCCTGA<br>CTGCATCATCAGCGAGAACA	This work

nemA	24	CTTTCAGTGGGGAATACAGT<br>GTCAGCGGTGCCATAAAAAT<br>CATCAACGGGGTAGGAATGT<br>GCACGTTGGCGATAGTATTC<br>CTTTTGCCTGGGCAGAAATT<br>AATTTGCTCCGGACTATGGA<br>GACCATTTTCAGCATGAACG<br>ATCGCCTGACCATTTTCATC<br>CGGCATGGATGTTTCAACAC<br>AATCTCTTCCAGTTCAAGCG<br>GCTCTACCAGATCAAAACCG<br>AAATAACCGTGAGCAGAGTG	AGGAGAAAGGAACTGATGCA<br>TACCAAACGTGCGCGATTTT<br>CATTCTTCAATCCCGGCATC<br>AACGCGAATGCCAATGCGAT<br>TCTGGAAAGTACCGATTGGT<br>TTCATTCGGGCCGTTATCTG<br>ATCAGATACAGTGCATCGGC<br>ATAAGCAATGCCGCGTTTAC<br>TTTGCCGATCAGCGTTTCAG<br>TGTGGGTTAAGCTCAGCTTT<br>ACCGTAGAAACTTTCGGCAC<br>ACGTCGGGTAATCGGTATAG	This work
metN	24	TGGTGGAACACTTTGGTGAT<br>GCACCGATAACGCCATAAAT<br>TATAAGCGTACTCTTACCCG<br>GCTCCAGCAGGTTTACACAA<br>TTGGTCAACTCGGATTCTGA<br>AAATCATACCAATCTGGCGG<br>AGAGCCACGTTGCCAAAAAC<br>GACGTTTGACCTCGTCTTTC<br>CAATGACAGCAATTCCGTCA<br>CAATTGCCACACGTTGTTTC<br>TTTGGGATTGCTGGCTAACG<br>TGGCTTCATCACACAGCAAT	GAATAGAACGTGTCGTTGCC<br>AACAGAATCGTCAACCCCAG<br>TTCACAACGTCCATTTCGTG<br>GAATAAACTTCTGCGCCAGC<br>AGACGTTCCTGGTAATCTTC<br>ACGCAGTCAGTAAATGGCTC<br>ATTGACCGGTAAACTCCAGA<br>TTCAGAAAGCAGTGGGGCAT<br>CTGCGCGCTAATAATGTTGT<br>CTTGCGTATCTTGTTGTGTG<br>TTTACATGGTGTTCCTGCAG<br>GACATAACCCAGTACCTCTA	This work
rho	24	TATTTTCGCCGAGAGTGATC<br>AATGTCCTGCTTACGCATAC<br>TATCTCCAGTACGCCATCAC<br>GGAAACCAAATCCATCCTGC<br>GGCGAATCTTACCAGAGATG<br>TTCGTTAACTTTCAGCAGCG<br>GCGGGGTTAAGTTCTCAAAG<br>AGTAGAACCGTTACCACGTT<br>GTACGCGAGCAGTTAAATCT<br>CATGGTTTTACCGGCTTTCG<br>ATGCTCTGAGCAATGTTCTG<br>TCGATCAGCAGAACCATCAG	TGCATCTCGGTTACTTCTTC<br>GTTCGTCAAAGGTAGAAGCA<br>CTTCTCGATCACCATTTCCG<br>AGTGATGGAGTCGAGCAGAA<br>AACAACGGTGTTGTAAGCGC<br>CACCAAAGAAGCGTTTCGGA<br>AGAACCGGTATCGATAAGCG<br>TTACGAGAGAGGTGCAGTTC<br>GAAGACGCGTTTTTCAGCGA<br>CAGCTCTTCTTTACGGGTAC<br>GTGAATGATTTTGCGCAGGA<br>TTCCATTGCATCGATTTCGC	This work

cspA	8	CGATACCAGTCATTTTACCG<br>TTGTCAGCGTTGAACCATTT<br>TCAGGAGTGATGAAGCCGAA<br>CGAACACATCTTTAGAGCCA	GTTCTGGATAGCAGAGAAGT<br>CGTCCAGAGATTTGTAACCA<br>GTGAAGGACACTTTCTGACC<br>TACAGGCTGGTTACGTTACC	This work
cI	30	GGTTTCTTTTTTGTGCTCAT<br>CTCAAGCTGCTCTTGTGTTA<br>AATTGCTTTAAGGCGACGTG<br>GGGATAAGCCAAGTTCATTT<br>ATCTTGTCTGCGACAGATTC<br>AATAAAGCACCAACGCCTGA<br>GCATTTAATGCATTGATGCC<br>TGCAAGCAATGCGGCGTTAT<br>CTTCAACGCTAACTTTGAGA<br>CTGGCGATTGAAGGGCTAAA<br>CGCTTCATACATCTCGTAGA<br>TAAGTGACGGCTGCATACTA<br>ACAGGGTACTCATACTCACT<br>CCCTGCCTGAACATGAGAAA<br>TTCTAAGCTCAGGTGAGAAC	TCCGCATCACCTTTGGTAAA<br>TTTGGTTGTGCTTACCCATC<br>AGAATGCAGAATCACTGGCT<br>CGGTCATGGAATTACCTTCA<br>AGCTTGGCTTGGAGCCTGTT<br>AGAATTAACATTCCGTCAGG<br>AACAGCCTGCTCAGGGTCAA<br>CTATGCAGAAATCACCTGGC<br>AACTCATCACCCCCAAGTCT<br>CCTGATCAGTTTCTTGAAGG<br>GTAAAAACACCTGACCGCTA<br>TTGGGTACTGTGGGTTTAGT<br>CAACTCTCATTGCATGGGAT<br>AGCGATAACTTTCCCCACAA<br>AAACGTCTCTTCAGGCCACT	95

Table S3: Sample sizes for smFISH datasets

Gene	Number of cells in smFISH dataset 1	Number of cells in smFISH dataset 2
dnaA	2701	1772
nrdA	1481	1203
nemA	1077	2582
metN	1370	1892
rho	2113	823
cspA	572	1772
cI (Negative control)	841	1309

Table S4: Evidence of repressed state in high-amplitude cell cycle expression clusters. Evidence that genes within *E. coli* clusters Ec9 and Ec17 (Fig. 5C & D) are autorepressed or otherwise in a repressed state. Besides the sources listed, the EcoCyc[77,96] database was used as a major source of information.

Gene ID	Gene name	Description	Cluster	Operon	Regulation/evidence of repression	Ref.
b3872	*yihL*	Putative DNA-binding transcriptional regulator	Ec17	*yihLM*	Nac-repressed; *yihL* is a GntR-family regulator so may have repressor function; *yihM* is induced by hexane so may have specific regulation	[97,98]
b4017	*arpA*	Regulator of acetyl CoA synthetase	Ec17	*arpA*	Unknown, but gene immediately downstream of the autorepressed transcription factor *iclR*	
b4018	*iclR*	DNA-binding transcriptional repressor IclR	Ec17	*iclR*	Autorepression (also represses aceBAK operon)	[99]
b4191	*ulaR*	DNA-binding transcriptional repressor UlaR	Ec17	*ulaR*	Regulation unknown but repressor of ulaG and ulaBCDEF operons	[100]
b4278	*insG*	KpLE2 phage-like element; IS4 putative transposase	Ec17	*insG*	Unknown but NanR repressor binds promoter	[101]
b1650	*nemA*	N-ethylmaleimide reductase	Ec17	*nemRA-gloA*	Operon autorepressed by NemR (*gloA* partially transcribed by read-through from this operon)	[35–37]
b3502	*arsB*	Arsenite/ antimonite:H+ antiporter	Ec17	*arsRBC*	Operon autorepressed by ArsR	[102]
b4014	*aceB*	Malate synthase A	Ec9	*aceBAK*	Repressed by IclR; repressed by CRP in the presence of glucose	[103]
b4015	*aceA*	Isocitrate lyase	Ec9	*aceBAK*	Repressed by IclR; repressed by CRP in the presence of glucose	[103]
b4016	*aceK*	Isocitrate dehydrogenase kinase/isocitrate dehydrogenase phosphatase	Ec9	*aceBAK*	Repressed by IclR; repressed by CRP in the presence of glucose	[103]
b2675	*nrdE*	Ribonucleoside-diphosphate reductase 2, α subunit dimer	Ec9	*nrdHIEF*	Repressed by NrdR; repressed by FUR in the presence of iron	[104,105]
b2676	*nrdF*	Ribonucleoside-diphosphate reductase 2, β subunit dimer	Ec9	*nrdHIEF*	Repressed by NrdR; repressed by FUR in the presence of iron	[104,105]
b3574	*plaR*	DNA-binding transcriptional repressor PlaR	Ec9	*plaR*	Autorepression (also represses L-lyxose catabolism operon)	[106]
b3605	*lldD*	L-lactate dehydrogenase	Ec9	*lldPRD*	Autorepression by LldR within the same operon	[107]
